# Saunders’s Medical Hand-Atlases: Atlas and Epitome of Human Histology and Microscopic Anatomy

**Published:** 1903-07

**Authors:** 


SAUNDERS'S MEDICAL HAND-ATLASES:

ATLAS AND EPITOME OF HUMAN HISTOLOGY AND MICROSCOPIC ANATOMY. By PRIVATDOCENT DR. J. SOBOTTA, of Wurzburg. Edited, with additions, by G. Carl Huber, M. D., Junior Professor of Anatomy and Histology, and Director of the Histological Laboratory, University of Michigan, Ann Arbor. With 214 colored figures on 80 plates, 68 text-illustrations, and 248 pages of text. Philadelphia and London. W. B. Saunders & Co. 1903. [Cloth, $4.50 net.]

As one follows out the list of medical hand-atlases published in this country by Saunders, one naturally wonders if there is any limit to the work. The latest of the books issued, admirably illustrates the fact that there is no subject which cannot be improved upon in some way or another when handled by a master.

There are so many works of histology that it was something of a daring venture to present a small volume on the subject; but in this work of Sobotta's, which is edited by Dr. G. Carl Huber, director of the histologic laboratory of the University of Michigan, the excellent text and the well-chosen illustrations make the volume typic of its general title; it is both a handbook and an atlas. The illustrations are especially well done, which is all the more remarkable when one considers the exquisite colorings and anatomic corrections of previous issues of this series.

There are 80 lithographic plates and between 60 and 70 plates made by photomechanic methods. It will be interesting to quote from the editor's preface concerning the method adopted to secure original illustration:

> The preparations were photographed under the same magnification as that under which they were drawn. The photographs were then used as a basis for the drawings, in that outline drawings, even to the finest detail, were traced on tracing paper; these outline drawings were then transferred to drawing paper. This method assures an exactness of magnification; by means of it distortion is avoided, and during the preparation of the drawing the photograph serves as a control picture.

It does not need a reviewer's say-so to make apparent the excellence and accuracy of this method. Additional value is lent to the work and especially the illustrations by the fact that all were made from sections of human tissue, obtained from the bodies of executed criminals within a few hours after death, making the specimens fresh and normal in every respect.

